# Cerebrospinal fluid α synuclein concentrations in patients with positive AD biomarkers and extrapyramidal symptoms

**DOI:** 10.1007/s00702-021-02351-x

**Published:** 2021-05-25

**Authors:** Izabela Winkel, Natalia Ermann, Agnieszka Żelwetro, Bożydar Sambor, Barbara Mroczko, Johannes Kornhuber, Bogusław Paradowski, Piotr Lewczuk

**Affiliations:** 1 Dementia Disorders Center of the Medical University of Wrocław, Ścinawa, Poland; 2 Department and Clinic of Geriatrics, Medical University of Wrocław, Wrocław, Poland; 3 Department of Psychiatry and Psychotherapy, Universitätsklinikum Erlangen, and Friedrich−Alexander Universität Erlangen−Nürnberg, Erlangen, Germany; 4 Interdyscyplinarne Studia Doktoranckie Uniwersytetu SWPS, II Wydział Psychologii, Wrocław, Poland; 5 Affidea Sp.Z O. O. Legnica, Legnica, Poland; 6 Department of Neurodegeneration Diagnostics, Medical University of Białystok, Białystok, Poland; 7 Department of Biochemical Diagnostics, University Hospital of Białystok, Białystok, Poland; 8 Department of Neurology, Medical University of Wrocław, Wrocław, Poland; 9 Department of Psychiatry and Psychotherapy, Lab for Clinical Neurochemistry and Neurochemical Dementia Diagnostics, Schwabachanlage 6, 91054 Erlangen, Germany

**Keywords:** α Synuclein, Alzheimer’s disease, Cerebrospinal fluid, Extrapyramidal symptoms, Biomarker

## Abstract

Extrapyramidal symptoms (EP) are not uncommon in Alzheimer’s Disease (AD); when present, they negatively influence the course of the disorder. A large proportion of AD patients shows concomitant Lewy bodies’ pathology post mortem. Total α Synuclein (αSyn) concentrations are frequently increased in the cerebrospinal fluid (CSF) of AD patients, but are decreased in Parkinson’s Disease (PD) and Dementia with Lewy Bodies (DLB). αSyn CSF concentrations in AD patients with EP (EP+) have not been reported so far. αSyn and the four Neurochemical Dementia Diagnostics (NDD) CSF biomarkers, (Aβ1-42, Aβ42/40, Tau, and pTau181), interpreted according to the Erlangen Score algorithm, were measured in patients with positive NDD results and presence of extrapyramidal symptoms (NDD + / EP+; *n* = 26), in patients with positive NDD results and absence of extrapyramidal symptoms (NDD+ / EP−; *n* = 54), and in subjects with negative NDD results (NDD−; *n* = 34). Compared to the NDD− controls (379.8 ± 125.2 pg/mL), NDD+ patients showed, on average, highly significantly increased CSF αSyn (519 ± 141.3 pg/mL, *p* < 0.01), but without differences between NDD+ / EP+ and NDD+ / EP− subgroups (*p* = 0. 38). Moderate but highly significant association was observed between concentrations of αSyn and Tau (r = 0.47, *p* < 0.01) and pTau181 (r = 0.65, *p* < 0.01). Adjusted for diagnoses, age, and sex, subjects with more advanced neurodegeneration on neuroimaging showed significantly lower αSyn concentrations (*p* < 0.02). In the setting AD versus controls, the area under the receiver operating characteristic (ROC) curve was 0.804 [0.712; 0.896] with the sensitivity and the specificity of 0.863 and 0.618, respectively. αSyn in AD patients does not differentiate between subjects with- and without EP. Its increased average concentration reflects probably neurodegenerative process, and is not specific for any pathophysiologic mechanisms. Further studies are necessary to explain the role of CSF αSyn as a potential biomarker.

## Introduction

In contrast to well established and generally accepted pattern of the cerebrospinal fluid (CSF) biomarkers for the diagnosis of Alzheimer’s Disease (AD), which includes decreased values of Aβ1-42 and Aβ42/40, and increased concentrations of Tau and pTau181 (Lewczuk et al. 2018; Hansson et al. 2019), the diagnostic role of α Synuclein (αSyn) in Parkinson's Disease (PD) and Dementia with Lewy Bodies (DLB) is still a matter of debate. There seems to be consensus in the literature that the total αSyn CSF concentrations are decreased in these synucleinopathies, compared to control subjects without neurodegeneration, but the acceptance of αSyn as a routine biomarker is hampered by suboptimal diagnostic accuracy, relatively large inter-center variation, and lacking consensus which of the isomers should be targeted (Mollenhauer et al. 2008; Parnetti et al. 2016; Simonsen et al. 2016).

Growing body of literature shows co-occurrence of the neuropathologic findings characteristic for synucleinopathies in a large proportion of autopsy confirmed AD patients (Arai et al. 2001; Hamilton 2000; Slaets et al. 2013). This is often associated with spatial co-localization of αSyn and Tau lesions (Lippa et al. 1998; Marui et al. 2000). Furthermore, significantly increased levels of the intracellular αSyn were reported in the inferior temporal lobe of the brains of AD patients without detectable DLB pathology (Larson et al. 2017).

These observations, taken together with the postulated role of αSyn in pathophysiology of AD (Twohig and Nielsen 2019), lead to the obvious question, if CSF αSyn could be regarded as a potential biomarker, particularly in differential diagnosis of AD versus PD/DLB. Indeed, several studies addressing this issue have been published (Simonsen et al. 2016); (Twohig and Nielsen 2019), but little is known about αSyn CSF concentrations in subjects with AD and concomitant PD comorbidity. This seems to be particularly relevant, considering that majority of the published reports shows that, compared to the relevant control groups, αSyn CSF concentrations are decreased in synucleinopathies but increased in AD.

Therefore, in this retrospective cross-sectional study we measured αSyn CSF concentrations in carefully selected AD patients whose clinical, neuroimaging, and neuropsychologic diagnoses were confirmed by the four core CSF AD biomarkers, and who either exhibited or did not exhibit extrapyramidal symptoms (EP). We contrasted the results with those of the control group consisting of patients without alterations in the core CSF AD biomarkers and without EP.

## Materials and methods

### Patients and the CSF collection, neuroimaging, and the core CSF biomarkers

The study was approved by the ethical committee at the Medical University of Wrocław, Poland. The patients were recruited at the Diagnostic and Research Center for Alzheimer’s disease, Ścinawa, Poland. The AD diagnoses were performed according to the NIA-AA criteria (McKhann et al. 2011), which was, however, treated only as an additional, supportive criterion on the top of the CSF-based categorization. The CSF samples were collected by lumbar punctures into polypropylene test tubes. Portions of ca. 250 µL for the NDD and αSyn analyses were immediately aliquoted and frozen at −80 °C. Aliquots were then shipped on dry ice to the Lab for Clinical Neurochemistry and Neurochemical Dementia Diagnostics, Erlangen, Germany, where the laboratory analyses were performed. In all cases, total protein concentration, glucose concentration, and cell count measured in the local laboratory. Samples contaminated with blood were not used for the study. In all subjects, the four core Neurochemical Dementia Diagnostics (NDD) CSF biomarkers were measured with ELISA: Aβ1-42, Aβ1-40 (IBL International, Hamburg, Germany), Tau, and pTau181 (Fujirebio Europe, Ghent, Belgium), according to the vendors' protocols. The results of the core biomarkers were interpreted according to the previously published and validated Erlangen Score (ES) algorithm (Lewczuk et al. 2009, 2015; Baldeiras et al. 2019; Skillback et al. 2019; Somers et al. 2019). The subjects with ES ≥ 3 were categorized as NDD+ patients (*n* = 80), and the subjects with ES ≤ 1 (*n* = 34) were categorized as NDD− controls. Furthermore, the NDD+ subjects were split into a subgroup with EP (NDD+ / EP + , *n* = 26), including: bradykinesia (in 10 subjects), muscle rigidity with cogwheel or lead-pipe muscle rigidity (in 6 subjects), concurrence of both (in 3 subjects), and asymmetric tremor (in 7 subjects), and subgroup without EP (NDD+ / EP− *n* = 54). Extrapyramidal symptoms did not cause pronounced motor and non-motor abnormalities and did not meet the criteria for atypical Parkinsonism. There were no episodes of psychotic productive symptoms in the study group. Neurologic conditions were assessed by two independent consultants experienced in neurogerontology.

In 101 subjects (70 NDD + patients and 31 NDD− controls), routine neuroimaging was performed with Signa HD XT 1.5 T scanner (GE Medical Healthcare, Chicago, IL, USA). For the evaluation of the structural alterations, Medial Temporal lobe Atrophy (MTA) scale was applied (Scheltens et al. 1992). The MTA-score is rated on the coronal T1-weighted images at a consistent slice position, in such a way that a slice through the corpus of the hippocampus is selected at the level of the anterior pons. The score is based on a visual rating of the width of the choroid fissure, the width of the temporal horn, and the height of the hippocampal formation. The score ranges from 0 (no atrophy) to 4 (severe volume loss of hippocampus). Results are age-dependent: for a person younger than 75 years a score of 2 or more is abnormal. For older patients, a score of 3 or more is considered abnormal. A high MTA-score is a very sensitive indicator to diagnose Alzheimer's disease and is present in the vast majority of patients. In controls a positive score is almost always absent. The evaluation of the MTA was performed entirely independent of the CSF biomarkers, by an experienced neuroradiologist who did not have access to the CSF data.

### α Synuclein assay

Concentrations of αSyn in the patients' CSF samples were assayed with a chemiluminescence method (Meso Scale Diagnostics; Rockville, MD, USA), according to the vendor's protocol. In preliminary experiments, four samples with different αSyn concentrations were pre-diluted with assay's buffer 1:4, 1:8, 1:16, and 1:32 to test for linearity and precision of the method. Resulting from this experiment, 1:8 dilution was chosen. All analyses were performed in duplicates. To test for inter-assay precision, a quality control (QC) sample of human pooled CSF was used.

### Statistical analysis

The number of subjects for the study was estimated under the condition of Satterthwaite's test for the populations with non-equal variances, with between-groups differences approximated according to the data in the literature (Mackin et al. 2015); the statistical significance and the power were set to 0.05 and 0.9, respectively. To avoid inconclusive results, in case we had obtained between-groups differences lower than those in the literature, the number of the study subjects was increased to 114.

If not stated otherwise, the results of the continuous variables are presented as averages ± standard deviations (SD) or 95% confidence intervals (95% CI). The results of the categorical variables are presented as percentages, and compared to one another with χ^2^ and/or Kruskal − Wallis test. Statistical analyses were used contrasting: (a) the total NDD+ group versus NDD− controls, with Helmert contrast, and (b) the NDD+ with EP (NDD+ /EP +) group versus NDD+ without EP (NDD+ / EP−) group. Correlations between continuous variables, unadjusted for other covariates, are presented as Pearson's correlation coefficients (r).

Associations between CSF αSyn concentrations and the disease categories, adjusted for explanatory variables, were analyzed with a series of linear regression models. First, a model with clinical− neurochemical categorization only was fitted (M0), subsequently supplemented with the demographic data (M1), supplemented further with the time of the disease duration and the MMSE test score (M2), and finally with the presence of the metabolic syndrome (diabetes mellitus type 2 and/or obesity) and the results of the MTA score (M3). If the numbers of the patients in the models were equal, the models were compared with the Likelihood Ratio test (LRT); otherwise the models were compared with the Akaike's Information Criterion (AIC). Taking into account the results of the models M0 − M3, in the final regression model (M4) age, sex, and MTA results were retained. Finally, interactions between the explanatory variables and the disease categorization variable were tested.

The αSyn concentration cut off, optimized for the separation of NDD− and NDD+ groups, was obtained at the maximized Youden index. The area under the receiver operating characteristic (ROC) curve (AUC) was calculated with nonparametric method. Logistic regression was used to model the sensitivity and the specificity as a function of covariates, according to Coughlin et al. (Coughlin et al. 1992), slightly modified by introducing an interaction term for covariates-by-disease status. The diagnostic accuracy was defined as the conditional probability of the agreement between the result of the α Syn test and the disease status, and was modelled, conditional on age, with logistic regression. If not stated otherwise, estimates in all models were obtained with the Maximum Likelihood method.

A *p* < 0.05 was considered significant. All statistical analyses were performed with Stata 14.2 (StataCorp, College Station, TX, USA).

## Results

### Demographic, neuropsychologic, neuroimaging, and the core CSF biomarkers results

Demographic and neuropsychologic data of the patients, and the concentrations of their core CSF biomarkers, are presented in Table 1. In one case of pTau181 and in three cases of Tau, unmeasurably low concentrations were obtained. For statistical analyses, these results were set to the lowest assays' standard concentrations (15.6 pg/mL and 40 pg/mL, respectively).

**Table 1:** Age, CSF biomarkers, and the MMSE score of the patients. Presented are averages and standard deviations

	NDD−	NDD+	NDD+ / EP−	NDD+ / EP +
	*n* = 34	*n* = 80	*n* = 54	*n* = 26
Age (yrs.)	64.5 ± 7.5	70.6 ± 8.6*	70.4 ± 8.2	71.0 ± 9.6
Aβ1-40	15,320 ± 4380	16,250 ± 5170	16,880 ± 4900	14,930 ± 5560
Aβ1-42	1021 ± 277	510.6 ± 135.7*	530.4 ± 138.7	469.5 ± 121.5
Aβ42/40	0.069 ± 0.018	0.033 ± 0.009*	0.033 ± 0.008	0.034 ± 0.011
Tau	204 ± 70.2	661 ± 322*	686 ± 366	608 ± 197
pTau181	42.5 ± 11.0	93.9 ± 23.2*	95.6 ± 23.4	90.4 ± 22.8
α Synuclein	379.8 ± 125.2	519 ± 141.3*	528.4 ± 151.0	499.5 ± 119.2
MMSE	24.7 ± 4.8	19.1 ± 5.1*	19.3 ± 5.3	18.8 ± 4.7

*significantly different (*p* < 0.05) compared to the NDD− control group

Briefly, NDD+ patients compared to the NDD− controls were on average significantly older, had expectedly lower concentrations of Aβ1-42 and Aβ42/40 ratio, higher Tau and pTau181 concentrations, and lower MMSE score. There were no statistically significant differences in Aβ1-40 concentrations between the two groups. The distributions of neither gender (*p* = 0.14) nor presence of the metabolic syndrome (*p* = 0.22) differed significantly between the two groups. None of the metrics differed significantly between the NDD/EP + and the NDD/EP− patients.

The distribution of the MTA scores is presented in Table 2. A non-significant tendency towards higher scores in the NDD+ patients compared to the NDD− controls (*p* = 0.054) was observed.

**Table 2:** Distribution of the MTA scores in the NDD+ patients and the NDD− controls. Presented are the numbers and the percentages of the total number per given group

	MTA score				
	0	1	2	3	4
NDD− (*n* = 31)	12 (39%)	12 (39%)	4 (13%)	3 (9%)	0 (0%)
NDD+ (*n* = 70)	11 (16%)	26 (37%)	19 (27%)	9 (13%)	5 (7%)

### α Synuclein assay performance

Four dilution factors (1:4, 1:8, 1:16, and 1:32) tested in the preliminary experiments resulted in practically the same concentrations (after taking the multiplication factor into consideration), as well as neglectable differences of the intra-assay imprecision (data not shown), reconfirming the linearity and the precision of the method. Median of the ranges-to-averages of the duplicate measurements of the 114 patients CSF samples was 2.1%, with inter-quartile range: 1.0–3.6%; CV of the average results of the QC sample in the four analytical runs (i. e. the inter-assay imprecision) was 7.6%.

### α Synuclein in the CSF; correlation with the core CSF biomarkers

**Fig. 1:**
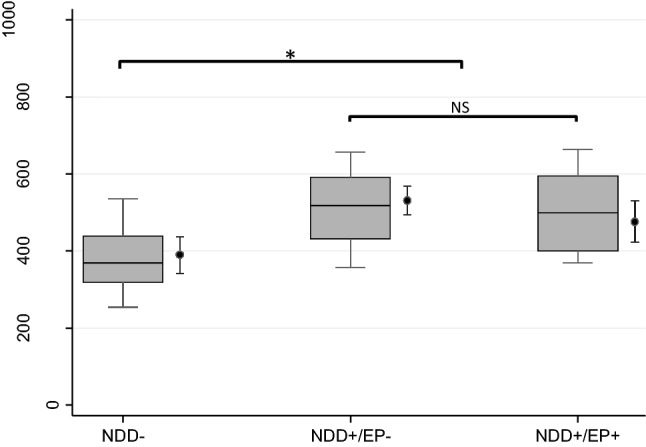
αSyn CSF concentrations in the three groups. Presented are: unadjusted medians (horizontal lines), 25–75 percentiles (boxes), and 10–90 percentiles (box whiskers). Right to the boxplots, adjusted linear marginal predictors (dots) with the corresponding 95% confidence intervals (dot whiskers) are presented post-estimated after the final regression model (M4)

We observed statistically highly significant difference between unadjusted average αSyn CSF concentrations in NDD+ patients and the NDD− controls (*p* < 0.01). We did not observe statistically significant difference between unadjusted αSyn CSF concentrations in NDD+ / EP+ and NDD+ / EP− patients (*p* = 0.38; Fig. 1). αSyn correlated moderately but significantly with age (r = 0.29, *p* < 0.01), Aβ1-40 (r = 0.42, *p* < 0.01), Aβ42/40 (r = −0.38, *p* < 0.01), Tau (r = 0.47, *p* < 0.01), and pTau181 (r = 0.65, *p* < 0.01), but not with Aβ1-42 (r = −0.16, *p* = 0.09).

**Fig. 2:**
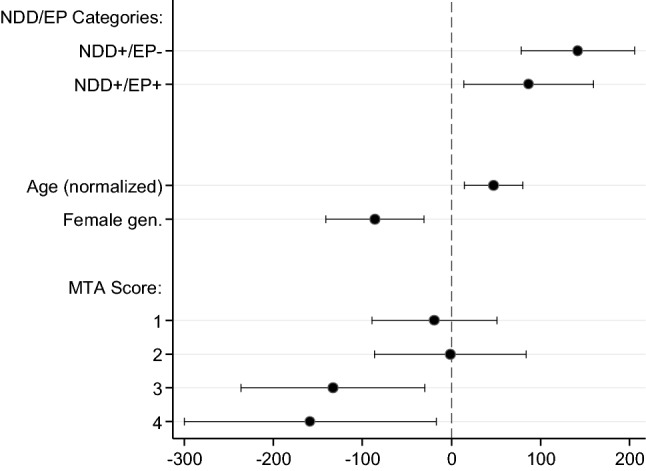
Regression coefficients (dots) and their 95% confidence intervals (spikes) from the final regression model (M4). For better visualization of the coefficient and CIs of age, the variable was normalized (divided by its standard deviation)

In the regression models M0–M3 (Table 3), we observed significant association of the CSF αSyn concentrations with patients categorization, age, sex, and the MTA score, adjusted for the covariates as indicated. We did not observe association of the αSyn CSF concentrations with the duration of the disease, the MMSE score, and presence of the metabolic syndrome. Hence, in the final model (M4) age, sex, and the MTA sore were retained. In this model, holding other covariates constant, NDD+ patients had significantly higher average αSyn concentrations than the NDD− controls (β = 114.2, *p* < 0.01), but there were no significant difference between NDD+ / EP + and NDD+/ EP− patients (β = −55.0, *p* = 0.11). Adjusted for other covariates, female gender (β = −85.8, *p* < 0.01), age (β = 5.43, *p* < 0.01), and MTA score (*p* < 0.02) were significantly associated with αSyn concentrations. Interestingly, keeping other covariates constant, patients with the MTA sore 3 (β = −132.9, *p* = 0.012), and the MTA score 4 (β = −158.4, *p* = 0.03) had lower average αSyn concentrations than those with the MTA score 0. We did not observe such association between higher MTA scores and alterations of the concentration of Tau, conditional on other covariates (data not shown). Regression coefficients from this model are presented in Fig. 2, and marginal predictions of the CSF αSyn concentrations resulting from this model are also presented in Fig. 1. We did not observe significant interactions between either age or sex and the NDD categorization of the patients in this model.

**Table 3:** Regression models testing the associations of the variables

Explanatory variable	Regression models				
	M0	M1	M2	M3	M4
	*n* = 114	*n* = 114	*n* = 113	*n* = 113	*n* = 101
Clinical–neurochemical categories^1^, compared to the reference category (NDD−):					
NDD+ / EP−	148.6 [89.3; 208.0]*	141.4 [80.7; 202.1]*	136.4 [68.0; 204.7]*	141.5 [69.6; 213.4]*	141.7 [78.0; 205.4]*
NDD+ / EP +	119.8 [49.1; 190.4]*	97.6 [26.4; 168.8]*	92.1 [13.2; 171.1]*	87.5 [3.9; 171.1]*	86.7 [14.1; 159.3]*
Age (yrs.)		3.5 [0.5; 6.5]*	3.5 [0.4; 6.6]*	5.4 [1.5; 9.3]*	5.4 [1.7; 9.2]*
Female gender		−62.7 [−115.4; −9.9]*	−65.4 [−119.8; −11.1]*	−86.5 [−143.8; −29.1]*	−85.8 [−140.9; −30.6]*
MMSE			−0.8 [−6.0; 4.4]	0.1 [−5.4; 5.6]	
Disease durat. (yrs.)			−3.2 [−13.9; 7.4]	−1.2 [−12.2; 9.8]	
Metabolic synd				−2.8 [−58.0; 52.3]	
MTA score^1^, compared to the reference category (MTA = 0):					
1				−18.0 [−91.4; 55.4]	−19.3 [−89.4; 50.8]
2				−0.5 [−88.6; 87.6]	−1.4 [−86.6; 83.7]
3				−131.6 [−240.1; −23.1]*	−132.9 [−236.2; −29.6]*
4				−154.5 [−304.1; −5.0]*	−158.4 [−300.1; −16.7]*
AIC	1448	1443	1435	1268	1274

^1^Coefficients of all categories are jointly significantly different from the corresponding reference category (Wald test)

### Sensitivity, specificity, and accuracy of α Synuclein as a potential AD biomarker

**Fig. 3:**
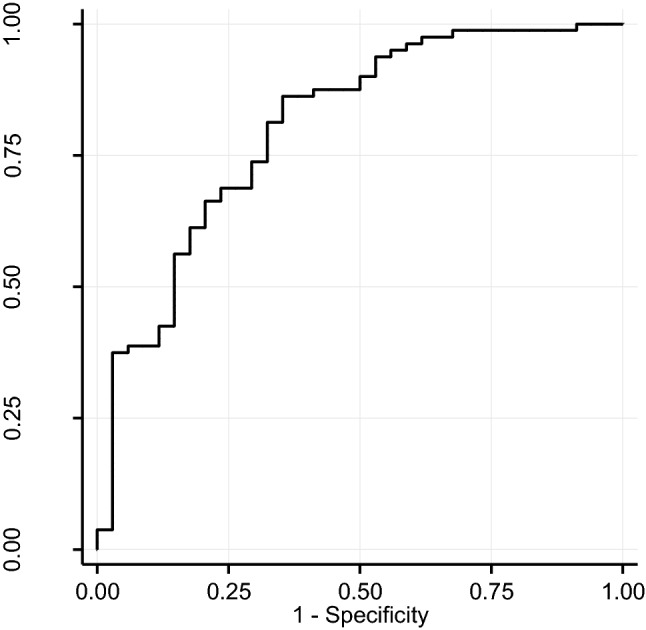
ROC curve in the setting NDD+ versus NDD−

ROC curve is presented in Fig. 3. The AUC was 0.804 [0.712; 0.896]. At the Youden point, maximizing the sum of the diagnostic sensitivity and the specificity separating the NDD+ patients from the NDD− controls, the cut off value of the αSyn concentration was 383 pg/mL. The sensitivity, the specificity, and the accuracy were 0.863 [0.787; 0.938], 0.618 [0.454; 0.781], and 0.798 [0.715; 0.864], respectively. In the logistic models, we did not find significant association of demographic data with the diagnostic metrics of αSyn (data not shown).

## Discussion

In the current study, we observed increased concentrations of αSyn in the CSF of patients with AD, whose diagnoses were supported by pathologic CSF biomarkers and neuroimaging findings, compared to the non-demented controls without alterations of the CSF AD biomarkers. We did not observe differences between the average CSF αSyn concentrations of AD patients with- and without EP. Weak, though significant, association between CSF αSyn and Aβ1-40 and Aβ42/40 was observed, with somehow stronger association with Tau and pTau181.

EPs are not uncommon in AD, with as much as 22.6% of community-dwelling AD patients presenting with them in the course of the disease (Portet et al. 2009). The frequency and severity of EP appear to increase over time along with AD severity (Ellis et al. 1996). Their clinical significance is poorly understood, and they probably result from different underlying mechanisms, and location or type of lesions, but clear association exists between presence and intensity of EP in AD subjects and more severe cognitive impairment, rapid cognitive decline, higher probability of institutionalization and higher socioeconomic burden (Portet et al. 2009).

A vast majority of studies published so far reports decreased CSF concentrations of total αSyn in PD subjects, compared to both healthy controls and/or patients with other neuropsychiatric disorders (a meta-analysis in (Eusebi et al. 2016)). On the other hand, large proportion of papers shows that CSF total αSyn concentrations tend to increase in AD compared to the controls (Twohig and Nielsen 2019). Increased αSyn concentrations in the AD subjects, along with moderate correlation between αSyn and Tau and/or pTau181 in the current study reconfirm the data and the conclusions reported by many investigators, that the increased CSF αSyn in AD seems to rather reflect unspecific neurodegeneration and not specific processes characteristic for AD (Oeckl et al. 2016; Korff et al. 2013; Slaets et al. 2014; Majbour et al. 2017). For example, Slaets et al. found a significant, moderately positive correlation between Tau and αSyn and between pTau181 and αSyn in AD patients but not in DLB patients (Slaets et al. 2014) Interestingly, not only association of αSyn with biomarkers of neurodegeneration (Tau) but also with biomarkers more specific for AD (Aβ peptides) was postulated; Buddhala et al. observed a positive correlation between CSF αSyn and Aβ1-42 in PD patients, but not in controls, confirming a pathophysiologic connection between the metabolisms of these proteins in PD (Buddhala et al. 2015). Taken together, considering that predominant source of αSyn in the brain are presynaptic neuronal terminals, it seems reasonable to hypothesize that degenerating neurons passively release αSyn molecules, which then diffuse to the CSF at increased rate.

Our cohorts were selected in such a way, that the results of the four AD CSF biomarkers (Aβ1-42, Aβ42/40 ratio, Tau, and pTau181) were considered the major categorization criterion, with the patterns of the CSF biomarkers interpreted according to the Erlangen Score algorithm (Lewczuk et al. 2009, 2015). We believe that the CSF biomarkers, once established, properly validated, and consequently controlled for quality, are the most objective, investigator-independent diagnostic tools, resistant to all kinds of bias linked to an investigators (perhaps suboptimal) experience. This approach, taken together with moderate correlation between αSyn and Tau and/or pTau181, might explain why αSyn sensitivity and specificity, in the setting AD versus controls, resulted in our study in somehow larger ROC AUC and better diagnostic accuracy than those metrics reported by (Korff et al. 2013), and comparable to those reported by (Majbour et al. 2017). We believe that in a study like ours, with CSF biomarkers treated as an important categorization criterion, good performance of αSyn as a potential biomarker is rather a side effect of its correlation with the biomarkers of neurodegeneration (Tau and pTau181) and not its individual characteristic. Therefore, the results of the ROC analysis we are reporting in this study must be treated exclusively as an additional statistics, perhaps helpful in comparison our results to those reported by others, and not as a claim that αSyn could be of use as a specific biomarker.

We did not observe difference in the average CSF concentrations of αSyn between AD patients with EP and AD patients without them. Since neuropathologic findings characteristic for AD and PD/DLB overlap in a large proportion of subjects diagnosed *post mortem* and the literature shows clear tendencies towards increased αSyn in AD versus decreased αSyn in PD/DLB, a question raises whether patients presenting with dementia due to AD and concomitant clinical PD symptoms would show the net increase, decrease, or perhaps lack of alterations of the CSF αSyn. Toledo et al. found hallucinations as strong predictor of coincident α Syn aggregation in AD patients in an autopsy-controlled study (Toledo et al. 2013). Similarly, Mackin et al. found decreased CSF αSyn in AD patients with hallucinations and, when adjusted for Tau concentrations, with longitudinal decline in performance of executive function, *i. e.* two clinical symptoms often observed in DLB, compared to the AD patients without these symptoms. They concluded that reduction in αSyn concentration in these subjects may represent an association between AD and DLB-like symptomatology (Mackin et al. 2015). On the other hand, however, they also found reduced αSyn concentrations in AD patients with accelerated decline of memory and language, which is difficult to explain, as these domains are not characteristic for DLB. Therefore, we believe that explanations other than Lewy bodies’ pathology for the presence of parkinsonism, such as EP, in AD must be considered. Such underlying mechanisms may include the presence of senile plaques in the putamen, caudate and substantia nigra, and the presence of neurofibrillary tangles in the substantia nigra (Liu et al. 1997; Burns et al. 2005).

Perhaps the most unexpected finding of our study is the decreasing average concentrations of αSyn, but not Tau, with advancing neurodegeneration process observed in neuroimaging (expressed as the MTA score), conditional on the diagnoses, age, and sex of the subjects. A simple explanation that more intense neuronal loss leaves behind less neurons capable to act as a source of αSyn is probably oversimplification, as such association was not observed for Tau. Hence, it might be speculated that the (passive) release of αSyn and Tau from degenerating neurons follows different patterns. Interestingly, Mackin et al., following their findings of the reduced αSyn CSF concentrations in AD patients with accelerated overall cognitive deterioration, also concluded that the CSF αSyn may be reduced as an effect of an overall neuronal or synaptic loss (Mackin et al. 2015).

Our study is not without limitations. The most serious one is probably lack of autopsy confirmation of the diagnoses and the corresponding classification of the pathologies. We tried to overcome this limitation by placing more pressure on the CSF-based grouping of the subjects. Furthermore, the control group was recruited predominantly from close relatives of the AD patients hospitalized in the Center; in many instances, the control subjects expressed concerns regarding their cognitive functions, obviously associated with the every-day life experiences of living in the proximity of a closely related ill person (such as spouse or parent). This most probably clarifies why we observed decreased MMSE score in some of these subjects, which merely reflects their afflicted mood and mild anxiety. We also need to stress that the CSF-based categorization is not without limitations. Lumbar puncture is often regarded as an invasive procedure, and the NDD biomarkers characterize with high, but not one hundred percent, accuracy. Therefore, we believe that the diagnosis, and the classification of subjects in research studies, always requires a multi-expertise team including clinical neurochemist, neuropsychologist, neuroradiologist, and—last but not least—an experienced clinician. Certainly more studies are necessary to confirm our findings.

## Abbreviations

αSyn: α Synuclein
Aβ1-40: Amyloid β peptide with the amino acid sequence 1 to 40
Aβ1-42: Amyloid β peptide with the amino acid sequence 1 to 42
Aβ42/40: Concentration ratio of the peptides Aβ1-42 and Aβ1-40
AD: Alzheimer Disease
AIC: Akaike’s Information Criterion
AUC: Area Under the [ROC] Curve
CI: Confidence Interval
CSF: Cerebrospinal Fluid
DLB: Dementia with Lewy Bodies
EP: Extrapyramidal Symptoms
ES: Erlangen Score
LRT: Likelihood Ratio Test
M: A statistical model
MMSE: Mini Mental State Examination
MTA: Medial Temporal Lobe Atrophy
NDD: Neurochemical Dementia Diagnostics
NIA-AA: National Institute on Aging—Alzheimer’s Disease Association
PD: Parkinson’s Disease
pTau181: Tau molecule phosphorylated at the amino acid position 181
QC: Quality control
r: Pearson’s Correlation Coefficient
ROC: Receiver Operating Characteristic
SD: Standard Deviation
